# Individual differences in associative learning

**DOI:** 10.3389/fpsyg.2014.00466

**Published:** 2014-05-20

**Authors:** Robin A. Murphy, Rachel M. Msetfi

**Affiliations:** ^1^Experimental Psychology, Behavioural Neuroscience, University of OxfordOxford, UK; ^2^Department of Psychology, University of LimerickLimerick, Ireland

**Keywords:** learning, associationism, computational psychopathology, conditioning (psychology), individual differences

This ebook represents the scientific contribution of over 30 individuals working in laboratories in 5 countries (Belgium, Netherlands, Spain, the United Kingdom and the United States), unified by the study of associative learning and individual differences. Individual differences have a central place in the study of psychology both historically and in the present day. It was frustrating then that when we conceived the idea of developing this Frontiers Topic, we knew personally that there was considerable work being conducted in various labs that could be characterized as the “associative learning of individual differences” but we were also confounded by the observation that there was no natural scholarly home for this work to be published. By its very nature this type of interdisciplinary work is at the frontiers and the borders between disciplines. The Frontiers publishing model offered us an excellent opportunity to bring this work together. By doing so we now have a current impression of the work and ideas being grappled with in 2014. As a representative sample of this work, we think this volume makes a bold statement and would function as a useful primer for the subject.

The study of individual differences was part of the nucleus of activity for the original pioneers who settled psychology and the malthusian growth and divergence that followed during psychology's development. The polymath Sir Francis Galton (1822–1911), for instance, a true psychological inventor, developed early psychometrics as well as the use of quantitative and statistical methods (such as the correlation coefficient and the normal distribution) for evaluating and understanding individual variation. He was well placed to make use of differences as a key psychological concept. He was a relative of Darwin and as such had a sympathetic perspective on the role that variation might play in psychological development. Despite his involvement in a range of related activities, as well as ideas that today are somewhat anti-ethical to scientific objectivity (e.g., ideas on eugenics for instance), his impact on current psychological thinking cannot be overestimated. He stood to understand psychological and individual personality variables and their relation to behavior and cognition.

This ebook owes much to Galton's quantitative approach, both in relation to the focus on differences but also on the idea that mathematical and computational principles might provide a scientific tool for understanding these differences. For Galton his quantitative tools were used for sorting or separating the perceived groups of individuals and understanding how they might relate, for instance with the use of concept of regression toward the mean. For associative learning, and its cousin connectionist modeling, the computational principles that have developed (for instance the Least Means Square method) provide parameters and principles for understanding the psychological differences. Models that have been developed to account for associative learning (e.g., Rescorla and Wagner, 1972; Sutton and Barto, 1981) provide explanations and descriptions for the development of acquired associations and as such these models make concrete the parameters upon which individuals might vary. Variation conceived here might be at the level of behavior as described in the papers of this volume, but the models are also used to identify neural circuits and substrates which must be the bases for the differences described.

The first three papers in this volume outline approaches to the computational analysis of individuals and provides a powerful case for how and why individual differences should be studied (Sauce and Matzel, 2013), and how the acquisition of a response or association might vary within an individual across a particular training experience (Glautier, 2013) or between individuals across the same experience (Byrom, 2013). These three contributions describe some of the elements underlying the complexity in thinking and quantitative modeling of the computational approach.

The remaining contributions highlight the variation in approaches and topics of inquiry that are amenable to this analysis. In the first, we read how differences in association and learning might be supported by memory processes and linked with differences in rumination (Joos et al., 2013) and then how differences in learning are expressed differentially over the life course (Robinson and Owens, 2013). The next set of papers describe work from the field of Computational Psychopathology asking diverse questions but with a related analytic framework. We consider the field of computational psychopathology as an important and viable method for understanding a range of human pathology. These topics are quite diverse, the study of human anxiety (Arnaudova et al., 2013) compulsive gambling (Orgaz et al., 2013), drug dependency (Torres et al., 2013) and neuroticism and personality (He et al., 2013) as well as attempts to address the physical and psychological effects and origins of nausea induced by chemotherapy treatment (Rodríguez, 2013). In all, this provides only a sample of the possible areas of human experience to which this analysis might be brought to bear and provides the reader (we hope) with a primer to excite the possibilities.

## Conflict of interest statement

The authors declare that the research was conducted in the absence of any commercial or financial relationships that could be construed as a potential conflict of interest.
